# Clinical Results of Total Hip Arthroplasty in Two Patients with Charcot Hip Joints due to Congenital Insensivity to Pain with Anhydrosis

**DOI:** 10.1155/2018/1743068

**Published:** 2018-01-31

**Authors:** Daisuke Inoue, Tamon Kabata, Yoshitomo Kajino, Tadashi Taga, Takashi Yamamoto, Tomoharu Takagi, Takaaki Ohmori, Hiroyuki Tsuchiya

**Affiliations:** Department of Orthopaedic Surgery, Graduate School of Medical Science, Kanazawa University, Kanazawa, Ishikawa, Japan

## Abstract

Traditionally, Charcot arthropathy has been considered an absolute contraindication for total hip arthroplasty (THA). However, some recent reports have shown that good short- to mid-term results can be achieved by improving the durability of the implant. This paper reports the mid- to long-term results of THA in two patients with Charcot hip joints caused by congenital insensivity to pain with anhydrosis. Both patients suffered multiple posterior dislocations in the six months immediately following surgery. However, with the continuous use of a hard abduction brace, one patient was eventually able to walk with a lofstrand cane and the other with the use of one crutch. Although one patient experienced a dislocation five years after surgery, X-rays taken after nine years and five years, respectively, revealed no clinical signs of implant loosening. We conclude that, with careful planning and appropriate precautions, THA may be a viable treatment option for Charcot hip joints caused by congenital insensivity to pain with anhydrosis.

## 1. Introduction

The term Charcot hip joint refers to a rapid and progressively destructive arthropathy resulting from various neurological conditions. The causes of Charcot hip joint include tabes dorsalis, syringomyelia, peripheral nerve injury due to diabetes mellitus, and congenital insensivity to pain with anhydrosis. Charcot arthropathy caused by these diseases has long been considered an absolute contraindication for total hip arthroplasty (THA) [1]. Recently, a few reports have shown good short- to mid-term results for THA, due to improving the durability of the implant [2–4]. In particular, a recent report by Erdil et al. describes their short-term results of total hip arthroplasty in patients with Charcot hip joints due to congenital insensivity to pain with anhydrosis [4]. But, to date there have been no reports describing the mid- to long-term results of total hip arthroplasty in such patients.

We have performed THA on two patients with charcot hip joints due to congenital insensivity to pain with anhydrosis. Therefore, the purpose of this study is to report the mid- to long-term clinical results of the procedure in these cases.

## 2. Report of the Cases

### 2.1. Preoperative Planning

This investigational protocol was conducted with the approval of our institutional ethical committee. In accordance with the requirements of this review, all patients were provided informed consent.

Both patients were given preoperative hip X-rays and CT scans so that we could conduct precise preoperative planning. The authors performed the preoperative planning using a CT-based navigation system (Stryker Inc., Mahwah, NJ) in both cases, as detailed in our previous study [5]. Stem anteversion was determined in accordance with preoperative femoral anteversion. In femoral offset, stem selection was made so as not to be insufficient compared with the contralateral side. On the acetabular side, the acetabulum component implantation site was located at the point where the original acetabulum contacted the lateral wall of the teardrop and achieved a cup CE angle of more than 0°. The target inclination or anteversion angle of the acetabular cup was determined as described by Widmer and Zurfluh [6]. The cup size was chosen so as to fill the anterior and posterior acetabular walls. A morselized bone graft was performed in the acetabular bone defect of the weight bearing portion.

### 2.2. Case 1

A 38-year-old female had suffered from congenital insensivity to pain with anhydrosis since birth. At age 27, she was admitted to our hospital due to swelling of the right hip joint without a cause. A fracture of the right femoral head fracture was observed in a hip X-ray, and she was diagnosed as having a Charcot hip joint because the collapse of the right femoral head worsened. We followed up this patient carefully, with a conservative treatment, because it was possible for her to walk on one crutch. When she was 29 years old, she suffered from Charcot spine; spinal fusion was performed six times due to collapse of the spinal joint. She was admitted to our hospital again because she could no longer stand, due to limb length discrepancy.

**Figure 1:**
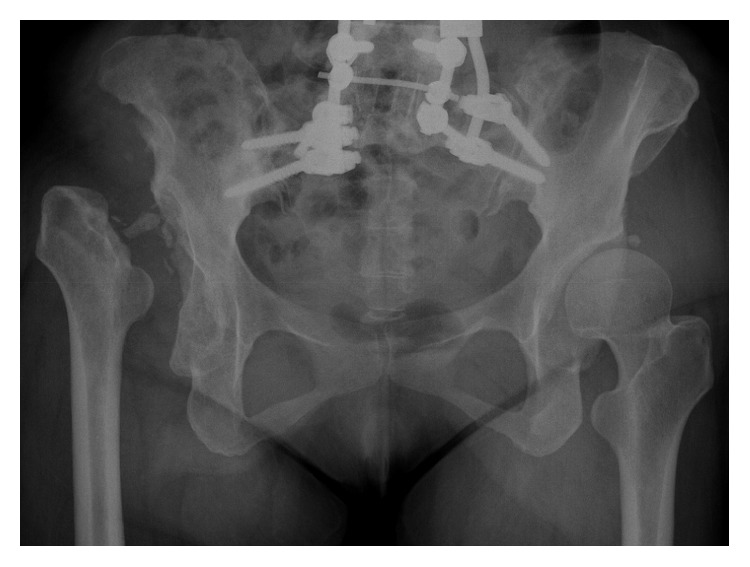
Case 1 preoperative hip X-ray. Right Charcot arthropathy of the hip shows typical radiographic changes.

Clinical examination revealed a swelling of the right hip joint and limping due to a shortening of the right hip joint, but the hip was almost pain free. A hip X-ray revealed the disappearance of the right femoral head, destructive changes in the right acetabulum, and a 36 mm shortening of the right hip joint (Figure 1).

We used a posterolateral approach, performed in the lateral decubitus position. On the acetabular side, we used medial protrusion technique because acetabular bone defect of the weight bearing portion was so severe [7]. The cementless acetabular cup was press fit in accordance with the preoperative plan. In addition, we inserted dome screws into the cementless acetabular cup in order to achieve initial fixation. On the femoral side, we performed a gentle rasping technique to prevent femoral diaphyseal fracture and inserted the cementless femoral implant into the femoral canal. After inserting the implant, we performed an impingement test to prevent bone-to-implant or bone-to-bone impingement. Finally, the short external rotator muscles separated from the proximal femoral bone were completely repaired to their original position. The postoperative rehabilitation schedule was full weight bearing after the operation; a hard abduction brace was used to prevent postoperative dislocation.

**Figure 2:**
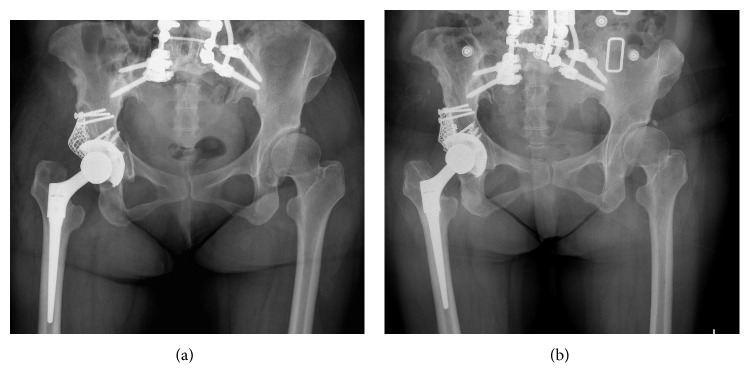
Case 1 postoperative hip X-rays. (a) Immediately after operation. (b) Nine years after operation. There were no clinical signs of implant loosening in the hip X-ray.

Two weeks after surgery, although she had been put in a hard abduction brace, postoperative posterior dislocation occurred when she pulled on her trousers and twisted her right hip joint. After closed reduction, we supervised the posture of dislocation and kept the patient in a hard abduction brace continuously. However, posterior dislocation occurred 5 times in the six months after surgery. Afterwards, with continuous use of the hard abduction brace, the posterior dislocation did not recur, and it was possible for her to walk with a lofstrand cane. Today, 9 years after surgery, the patient can walk with the lofstrand cane without causing implant dislocation, although it had been impossible for her to walk before surgery; X-rays revealed no clinical signs of implant loosening compared with the postoperative X-rays (Figure 2).

### 2.3. Case 2

This patient, a 38-year-old male, is the Case 1 patient's younger brother who also suffered from congenital insensivity to pain with anhydrosis from birth. He suffered from Charcot spine, and spinal fusion was performed many times due to the collapse of the spinal joints. During this treatment, hip X-rays showed the collapse of left femoral head getting worse, and we followed up this patient carefully, using conservative treatment, as long as it was possible for him to walk on one crutch. However, he was admitted to our hospital suddenly because he felt a swelling in his right hip joint and then could no longer stand.

**Figure 3:**
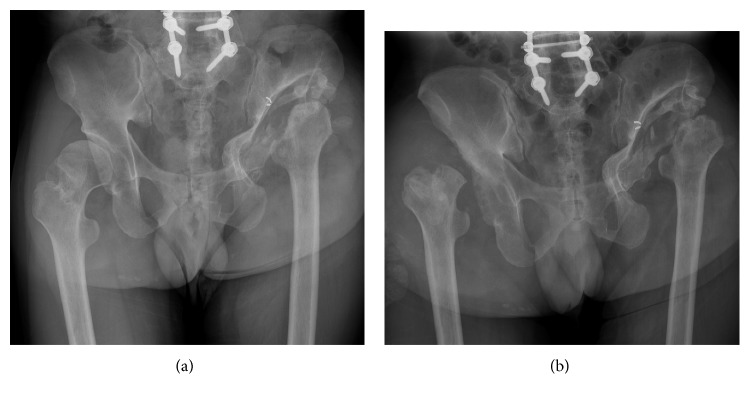
Case 2 preoperative hip X-rays. (a) Two months before admittance to our hospital. (b) Preoperative hip X-ray. A hip X-ray revealed the disappearance of right femoral head and destructive changes in the right acetabulum.

Clinical examination revealed swelling and a shorting of the right hip joint, but the joint was completely pain free. A hip X-ray revealed the disappearance of the right femoral head and destructive changes in the right acetabulum; nevertheless, the right femoral head was not collapsed before two months (Figure 3). If this patient had not received THA, he would surely have spent the rest of his life in a wheelchair due to the collapse of the bilateral hip joints.

We used a posterolateral approach, performed in the lateral decubitus position with the same operation protocol of Case 1. In this case, the cementless acetabular cup was press fit in accordance with the preoperative plan. In addition, we inserted dome screws into the cementless acetabular cup in order to achieve initial fixation. On the femoral side, we inserted the cementless femoral implant into the femoral canal with the same operation protocol of Case 1. After inserting the implant, we performed an impingement test to prevent bone-to-implant or bone-to-bone impingement. Finally, the short external rotator muscles separated from the proximal femoral bone were completely repaired to their original position. The postoperative rehabilitation schedule was full weight bearing after the operation; a hard abduction brace was used to prevent postoperative dislocation.

**Figure 4:**
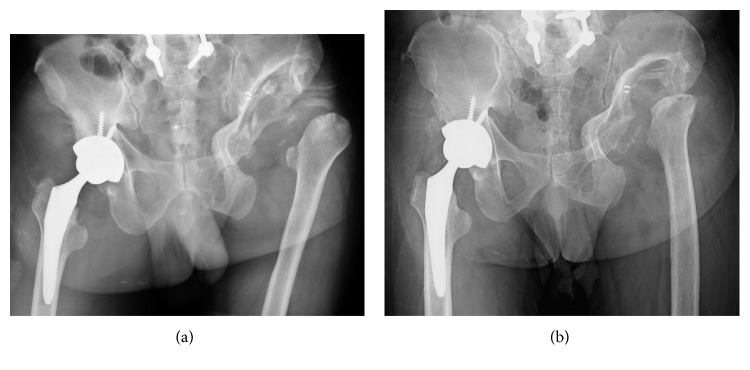
Case 2 postoperative hip X-rays. (a) Immediately after operation. (b) Five years after operation. There were no clinical signs of implant loosening in the hip X-ray.

Four weeks after surgery, despite being put in a hard abduction brace, postoperative posterior dislocation occurred, as in Case 1. Similarly, posterior dislocation occurred 3 times in the six months after surgery. Afterwards, through continuous use of the hard abduction brace, the posterior dislocation did not recur, and it was possible for him to walk on one crutch. However, five years after surgery, a late dislocation occurred when he arose from a chair. After closed reduction, we closely supervised the posture of dislocation and put him in a hard abduction brace continuously. There were no clinical signs of implant loosening in the hip X-ray taken, compared with the postoperative X-rays (Figure 4).

## 3. Discussion

Charcot hip joint refers to a destructive hip arthropathy caused by tabes dorsalis, syringomyelia, peripheral nerve injury due to diabetes mellitus, congenital insensivity to pain with anhydrosis, and so on. In the cases reported here, Charcot hip joint was caused by congenital insensivity to pain with anhydrosis. This rare neurological disorder is caused by a tyrosine kinase type NGF receptor deficiency of the autosomal recessive inheritance [8]. The NGF, which is a neurotrophic factor, does not activate, and both sensory and autonomic neurons are affected [9]. As a result, it is a clinical condition which causes systemic analgesia and adiaphoresis. Because pain is a protective sensory mechanism, the deficiency causes repeated minor traumas, and joint function is destroyed by severe joint swelling and repeated fractures. Robb et al. showed many complications after THA such as dislocation, aseptic loosening, or breakage of the implant in the early postoperative period because patients with this condition have inadequate muscle tension and complex femoral and acetabular deformities including excessive femoral anteversion, coxa valga, and leg-length inequality [10]. They reported one patient with Charcot arthropathy of the hip who suffered multiple postoperative dislocations after THA and finally was treated with component removal. However, we think that if THA is not performed, patients eventually become wheelchair-bound, with a decline in the quality of their daily lives.

A few papers have reported good results of THA for Charcot hip joints by improving the durability of total hip arthroplasty. Rapała and Obrebski reported on two patients with tabes dorsalis; their follow-up at 10 and 9.5 years confirmed the success of the THA, although one patient suffered two atraumatic postoperative dislocations [3]. Erdil et al. reported good short term results of cementless THA on one patient with congenital insensivity to pain with anhydrosis [4]. THA with congenital insensivity to pain with anhydrosis was first described in this report. Kraay and Bigach reported that implant design factors contributed to the reduction of postoperative risks and complications in total hip arthroplasty for neuromuscularly challenged patients [1]. In accordance with these past reports, we took various measures to prevent as many postoperative complications as possible when we performed THA in these patients. First, we used large femoral heads to reduce the risk of postoperative dislocation, with highly cross-linked polyethylene liners to reduce polyethylene wear. Next, we used CT-based three-dimensional templating software to select a femoral stem which achieved appropriate stem anteversion and femoral offset [5]. Third, we performed the surgery utilizing a CT-based navigation system to reproduce preoperative planning as closely as possible [11]. Finally, because early ingrowth failure or loosening is more likely to occur in these cases, we used supplementary screw fixation with the acetabular component to achieve initial fixation [12]. As a result, although early postoperative dislocation occurred in all cases due to sensory loss, deficiency of muscle tension, and lack of lumbar vertebrae compensatory change with the spinal fusion, neither patient showed clinical signs of implant loosening at the final long-term follow-up. One patient (Case 1) can walk without causing late dislocation even though it was impossible for her to walk before surgery, and the patient has restored normal hip function. In the other patient (Case 2), although late dislocation occurred when he rose from a chair, a hip X-ray showed no clinical signs of implant loosening. If the late dislocation is repeated, revision hip arthroplasty will be performed using a dual mobility or constrained acetabular component to reduce potential instability. Because dual mobility or constrained acetabular components have the possibility of reducing postoperative dislocation or joint instability, using this system appears to be reasonable option, although prosthetic dislocation, osteolysis, or aseptic implant loosening could occur in future, especially in these neuromuscularly challenged patients. Patients with congenital insensivity to pain with anhydrosis may appear to be at high risk of postoperative complications. However, it is certainly possible for these patients to regain normal hip function and the ability to walk. Thus, THA may be a viable treatment option for Charcot hip joints caused by congenital insensivity to pain with anhydrosis.

## 4. Conclusion

We have reported the mid- to long-term results of two patients who underwent THA for Charcot hip joints due to congenital insensivity to pain with anhydrosis. Although both patients experienced early dislocation, obvious implant loosening or breakage was not observed in either case over the mid- to long-term. THA thus may well be a viable treatment option for Charcot hip joints caused by congenital insensivity to pain with anhydrosis.
